# Plant Fertilization Interacts with Life History: Variation in Stoichiometry and Performance in Nettle-Feeding Butterflies

**DOI:** 10.1371/journal.pone.0124616

**Published:** 2015-05-01

**Authors:** Hélène Audusseau, Gundula Kolb, Niklas Janz

**Affiliations:** 1 Department of Zoology, Stockholm University, Stockholm, Sweden; 2 Department of Ecology, Environment and Plant Sciences, Stockholm University, Stockholm, Sweden; Evolutionary Biology Centre (EBC), Uppsala University, SWEDEN

## Abstract

Variation in food stoichiometry affects individual performance and population dynamics, but it is also likely that species with different life histories should differ in their sensitivity to food stoichiometry. To address this question, we investigated the ability of the three nettle-feeding butterflies (*Aglais urticae*, *Polygonia c-album*, and *Aglais io*) to respond adaptively to induced variation in plant stoichiometry in terms of larval performance. We hypothesized that variation in larval performance between plant fertilization treatments should be functionally linked to species differences in host plant specificity. We found species-specific differences in larval performance between plant fertilization treatments that could not be explained by nutrient limitation. We showed a clear evidence of a positive correlation between food stoichiometry and development time to pupal stage and pupal mass in *A. urticae*. The other two species showed a more complex response. Our results partly supported our prediction that host plant specificity affects larval sensitivity to food stoichiometry. However, we suggest that most of the differences observed may instead be explained by differences in voltinism (number of generations per year). We believe that the potential of some species to respond adaptively to variation in plant nutrient content needs further attention in the face of increased eutrophication due to nutrient leakage from human activities.

## Introduction

Stoichiometry—the availability and balancing of the chemical elements that make up living organisms—has been shown to influence a wide range of processes, from the function of the cellular machinery to population dynamics, ecological succession and ecosystem structure [1–4]. Work done on stoichiometry has mainly focused on the relationship between carbon, nitrogen and phosphorous (C:N:P), because of their importance as energy source and as basic building blocks in many essential molecules of life, such as proteins, nucleic acids, lipids, etc. [1, 5].

Studies have shown that optimal stoichiometric relationships are not static but vary both within and between species [6] and environments [7], but also between developmental stages [8, 9]. In addition, the availability of these nutrients influences fundamental life-history trade-offs and allocation patterns [10, 11]. For example, the available levels of nutrients in a given environment or food type constrain the maximum growth rate [1, 12, 13]. Conversely, the Growth Rate Hypothesis states that individuals that grow fast will be more likely to be P-limited, as the increased protein synthesis in rapidly growing organisms requires allocation to P-rich ribosomal RNA [1, 2]. Thus, at the scale of an organism, a basic challenge is to acquire adequate amounts of nutrients to support growth, development and reproduction from resources that seldom match their own stoichiometry [14].

In plant-feeding insects, the problem of achieving a balanced intake of necessary nutrients is particularly pronounced, partly because they are much more nutrient-rich than their food, but also because the nutritional content can differ substantially between plants [14–16]. Differences in soil nutrient levels [4, 13, 17] will affect plant nutrient content and this in turn may influence nitrogen—or phosphorous—limitation as well as life histories of the insects that feed on them [18, 19]. One defining characteristic of plant-feeding insects that has far-reaching consequences is the degree of host specificity (e.g. [20]). Although a majority of these insects are highly specialized, there is a full spectrum of variation among them, from strictly monophagous to highly polyphagous [21–23].

In terms of stoichiometry it would seem that a generalist has an advantage in that it should be able to achieve a balanced nutritional intake that matches organismal requirements by adaptive selection of available food items. However, diet-mixing is not necessarily advantageous from the perspective of feeding efficiency [24], and moreover, many polyphagous larvae are functionally specialists, as they normally complete their development on one host individual. Such generalist larvae share the problem of achieving a desired nutritional level and balance from the host plant chosen with specialists, but generalist larvae face the added complexity of maintaining a capacity to develop on a range of different plant species with potentially different nutrient content.

It appears reasonable that it should be more difficult for a generalist to fine-tune metabolization, consumption rate and growth strategy to the host, when they have several hosts that differ in chemical composition. Still, the existence of a general trade-off between feeding performance and the ability to feed on multiple hosts is controversial. While some studies have demonstrated such trade-offs (e.g. [25–27]), the majority of studies have, perhaps surprisingly, found weak or positive correlations in performance across hosts (e.g. [28–31]). However, it is possible that a difficulty to efficiently utilize multiple hosts will manifest itself on a more subtle level, such as in the ability to respond adaptively to intraspecific variation in plant nutritional state. A high-nutrition plant individual offers the possibility to regulate growth to optimize development, and adult size, but potentially also to achieve higher feeding efficiency, so that they can consume less for the same unit growth [15]. Such a growth strategy—that is fine-tuned to the nutritional status of the host plant—may be more difficult for a host plant generalist, which may respond differently to stochiometric variation. Moreover, even if nitrogen and phosphorus are often limiting nutrients, there is evidence that too high levels of nutrients can also be problematic [32, 33], and presumably, the ability to deal with very high nutrient levels requires specialized adaptations.

To our knowledge no study has so far experimentally investigated interspecific differences in life history traits (here we focused on the degree of host plant specialization), species sensitivity to food stoichiometry and their consequences in terms of fitness, even though a recent correlative study at a large spatial scale has suggested such correlations [34]. Therefore, in this study, we propose to look at three nymphalid butterflies that all share the same host *Urtica dioica* (stinging nettle). This plant has a predominantly Holarctic distribution and is especially common in northern Europe. It is found in a diverse range of habitats, but it is known for its ability to thrive in nutrient-rich soils [35]. It is also a common weed connected with nutrient leakage around houses, farms etc. Because of this tolerance to extremely nutrient-rich soils, it should provide a high-nutrient resource for insects that are capable of taking advantage of it. Due to the high levels of fertilization associated with industrialized farming and an increased nitrogen deposition, levels of nutrients in the soil are in general increasing. However, it is likely that nutrient levels and C:N:P stoichiometry will vary considerably between growing sites of *U. dioica*.

The three butterfly study species show both differences and similarities in their life history. *Aglais urticae* and *Aglais io* (previously named *Inachis io*, [36]) are specialists, feeding almost exclusively on this plant, while the third species *Polygonia c-album* is polyphagous, capable of feeding on seven plants families (Urticaceae, Ulmaceae, Cannabaceae, Salicaceae, Betulaceae, Corylaceae, and Saxifragaceae, [37]). The three species also differ in phenology and patterns of voltinism. In the study area of southern Sweden, *A. urticae* emerges from diapause in early spring, and flies in two consecutive generations before hibernation, while *A. io* and *P. c-album* emerge later and are strictly univoltine at this latitude. Moreover, *P. c-album* lays eggs singly whereas *A. urticae* and *A. io* lay eggs in clutches.

The main objective of the study was to investigate how larval performance (development time, pupal mass and survival) of the three species was affected by induced variation in plant stoichiometry, and to what extent the differences can be understood in terms of differences in life history. We hypothesized that the performance of the two nettle-specialists should be more fine-tuned than the generalist to the nutritional status of their single host plant. To further investigate the mechanisms behind the variation in larval performance, we also analyzed body content of nutrients in the larvae. In general, we expected larval stoichiometry to reflect the nutrient content of their food. However, larvae may adjust their feeding behavior to keep their homeostasis, as a response to both nutrient excess and limitation, which would affect performance negatively [33]. In such a case of stoichiometric imbalance, we would instead expect body nutrient content to be stable across treatments.

## Materials and Methods

### Origin of butterflies and egg collection

Mated females of *Polygonia c-album*, *Aglais urticae* and *Aglais io* were wild-caught late April in five localities in the Stockholm area (University: 59°21′36.6″N, 18°04′01.7″E; Järvafältet: 59°26′10.2″N, 17°54′14.3″E; Tyresö: 59°14′03.0″N, 18°18′11.2″E; Margretelund: 59°28′25.5″N, 18°21′31.0″E; Domarudden: 59°31′02.1″N, 18°20′49.2″E) and then placed in cages (about 50*50*50 cm) under 7L:17D photoperiod and a temperature fluctuating around 25°C. Females were provided with food (sugar water) and host plants to allow oviposition. Humidity was maintained by covering the cage floor with moist paper towels. No specific permission was required for the experiments or for collecting insects and plants in any of the localities used in the study, as they fall under the Swedish Right of Public Access. None of the species are endangered or protected.

We collected eggs daily in a slightly different manner for each of the species. *P. c-album* females were placed individually in cages and single eggs were collected. *A. urticae* and *A. io* females were placed in cages in groups and we collected clutches. We collected eggs from seven adult females of *P. c-album* and used 12 clutches of *A. urticae* coming from at least five different females (5 cages), and 7 clutches of *A. io* coming from at least three different females (3 cages). Eggs were saved in 5 ml plastic containers before hatching under natural light and room temperature.

### Plant fertilization and leaf sampling

In the surroundings of the Stockholm University campus, plants from several *U. dioica* clones were dug out and potted in commercial sowing soil during late fall 2011 and spring 2012. Plants were reared outside and placed in the greenhouse to stimulate growth two to four weeks before to be used as food for larvae. Plants were randomly assigned to one of four treatments: control, nitrogen fertilization (+N), phosphorus fertilization (+P), and nitrogen-phosphorus fertilization (+N+P). Before fertilization, all plants were allowed to root for at least four weeks after they were potted. In the control treatment plants were watered once a week with 500 ml 0.5 ml/1l Rika (industrial fertilizer), to stimulate a minimal growth, and once a week with 500ml pure water. Plants in the nitrogen fertilization treatment were watered twice a week with 3g *NH*_4_ *NO*_3_ solved in 500ml water. Plants in the phosphorus treatment were watered twice a week with 1g *Na*_2_ *HPO*_4_ solved in 500ml water. Plants in the nitrogen-phosphorus treatment were watered twice a week with 3g *NH*_4_ *NO*_3_ and 1g *Na*_2_ *HPO*_4_ solved in 500ml water.

Leaves in each plant fertilization treatment were collected and saved for nutrient analyses (C, N, P). They were collected at intervals varying between 3 to 5 days over the rearing period. Moreover, leaves were also collected from 36 locations where larvae have been found feeding in the field. All leaf samples were dried and ground before the analyses. We aimed with these four different fertilization regimes to induce differences in plant stoichiometry in terms of N:P ratio (all ratios calculated by mass) between fertilization treatments.

### Larval rearing and sampling

Upon hatching, 629 eggs of *A. urticae*, 476 eggs of *P. c-album*, and 441 eggs of *A. io* were assigned to a fertilization treatment. Larvae were reared individually in small cups at constant temperature (20°C) and light cycle (12L:12D). Larvae were fed ad libitum with young leaves replaced daily or every second day. Leaf quality was maintained using a moistened sponge in the bottom of the cup.

During larval development we recorded date and mass of pupation, and survival. Sex was determined at the pupal stage. Moreover, samples of fifth-instar larvae and pupae in each fertilization treatment and for each species were frozen and saved for body nutrient analysis (we initially aimed for 10 larvae and 10 pupae of each species in each fertilization treatment). Prior to the analysis, samples were freeze-dried and ground.

### Nutrient content analysis

Phosphorus content (%P, dry mass basis) was assayed using persulphate digestion and ascorbate-molybdate colorimetry [38]. Nitrogen and carbon content (%N, %C, dry mass basis) was assayed in an Isotope Ratio Mass Spectrometer type *Europa integra* or an elemental analyzer. In this analysis, samples were oxidized and reduced to *CO*_2_ and *N*_2_, respectively, which were measured with a thermal conductivity detector and IR-detection. For the phosphorus analyses we used 3–4 mg material and for the carbon and nitrogen analyses we used 1–3 mg dry-weight material.

### Statistical analyses

All statistical analyses were performed in the statistical program R, version 3.0.2 [39]. Model fitting selected for the fixed factors following a backward elimination procedure. Models comparison was performed using the anova function and the Akaike Information Criterion (AIC) as a guideline. In the case of linear mixed-effect models, AIC was calculated using maximum likelihood [40]. The fit of the models retained was estimated according to the distribution of the residuals.

We first investigated if fertilization treatments induced the expected variation in plant stoichiometry. We tested for the effect of fertilization treatment (control, +N, +P, +N+P), time, and their interaction on leaf N:P ratio using an ANOVA followed by Tukey’s tests (initial model written as plant N:P ratio ∼ fertilizer*time). Time corresponds to the plant sampling date and was included to check that the leaf N:P ratio in each fertilization treatment was constant through time. The comparison of plants in the four fertilization treatments and plants sampled in nature was performed using a non-parametric ANOVA (Kruskal-Wallis) followed by a Wilcoxon pairwise comparison with Holm-Bonferroni correction as the variance found in N:P ratio of plants in nature was particularly large and no transformation could satisfy the assumptions of a parametric test. Additionally, in order to better understand the observed differences in plant N:P ratio between the four fertilization treatments, we analyzed the effect of fertilization treatment on leaf absolute nitrogen and phosphorus content independently. The details of these statistical procedures and their results are presented in S1 Text and S1 Appendix.

Next, we analyzed the role of food stoichiometry, as determined by plant fertilization treatments (control, +P, +N, +N+P), on body nutrient content. These analyses aimed to test for stoichiometric imbalance that could predict larval performance. For each larval stage (fifth-instar larva and pupa) we investigated the effect of plant fertilization treatment, species, their interaction, and included start date, on body N:P ratio using ANOVAs followed by Tukey’s tests (initial models written as body N:P content ∼ fertilizer *species + start date). We included start date (which corresponds to the date neonate larvae were placed in one of the treatments) to capture part of the potential temporal variation in food quality between early and late larvae. Again, we concentrated our analyses and interpretation on the effect of plant fertilization treatment on body N:P ratio. However, for the same reasons as for the analyses of plant stoichiometry, we also analyzed and discussed the relative changes in absolute body content of nitrogen and phosphorus independently. The details of the statistical procedure for these latter analyses and their results are presented in the Supporting Information (S1 Text, S2 Appendix, S3 Appendix, S2 Text, S4 Appendix, S1 Fig). For these analyses, the sample sizes were low and in order to not have to account for relatedness between individuals, which would have involved an extra parameter to be estimated from the model and an associated decrease of the power of the analysis, individuals were collected according to a stratified random sampling (relative to clutches and females, larval stage, fertilizer treatment and species). Moreover, three obvious outliers that considerably affected the assumption of homogeneity of the variances were excluded from the analyses of body N:P ratio in fifth-instar larvae. The outliers are likely due to non-homogeneous samples used in the analysis of body content of nitrogen and phosphorus. The effect of plant fertilization treatment on fifth-instar larval body N:P ratio with and without those outliers were similar.

We investigated the effect of plant fertilization treatment on survival rate following the method proposed by Crawley [41]. We performed generalized linear models using quasibinomial distribution to account for overdispersion. Our response variable was a two columns matrix binding together the number of individuals that survived and the number of dead individuals per family and species. We tested for the effect of plant fertilization treatment, species, and their interaction on survival rate (initial model written as [survived,dead] ∼ fertilization*species).

Finally, we investigated the effect of food stoichiometry on larval development time to pupation and pupal mass using linear mixed-models. We used the lmer function in the R package lme4 [42]. We built individual models for the measures of development time and pupal mass for each species in order to investigate species-specific responses to plant fertilization treatments. Moreover, because we were interested in comparing the effect of fertilization treatment on larval performance regardless of species-specific differences in life history, we standardized variation in each species to the mean development time to pupation and pupal mass of larvae of the same species feeding on the control treatment. In other words, the response variables of our models correspond to a measure of deviation in percentage: how much faster or slower larvae developed, and how much larger or smaller pupae were, compared to the mean value of larvae of the same species feeding in the control treatment. We investigated the effect of fertilizer, sex, their interaction, and we included start date (initial model written as fixed ∼ fertilizer*sex + start date). Larval family for *P. c-album* and larval clutch for both *A. urticae* and *A. io* were included in the models as a random factor to control for the variance due to differences in relatedness among individuals (initial model written as random ∼ 1∣family or 1∣clutch).

## Results

### Plant stoichiometry

**Fig 1:**
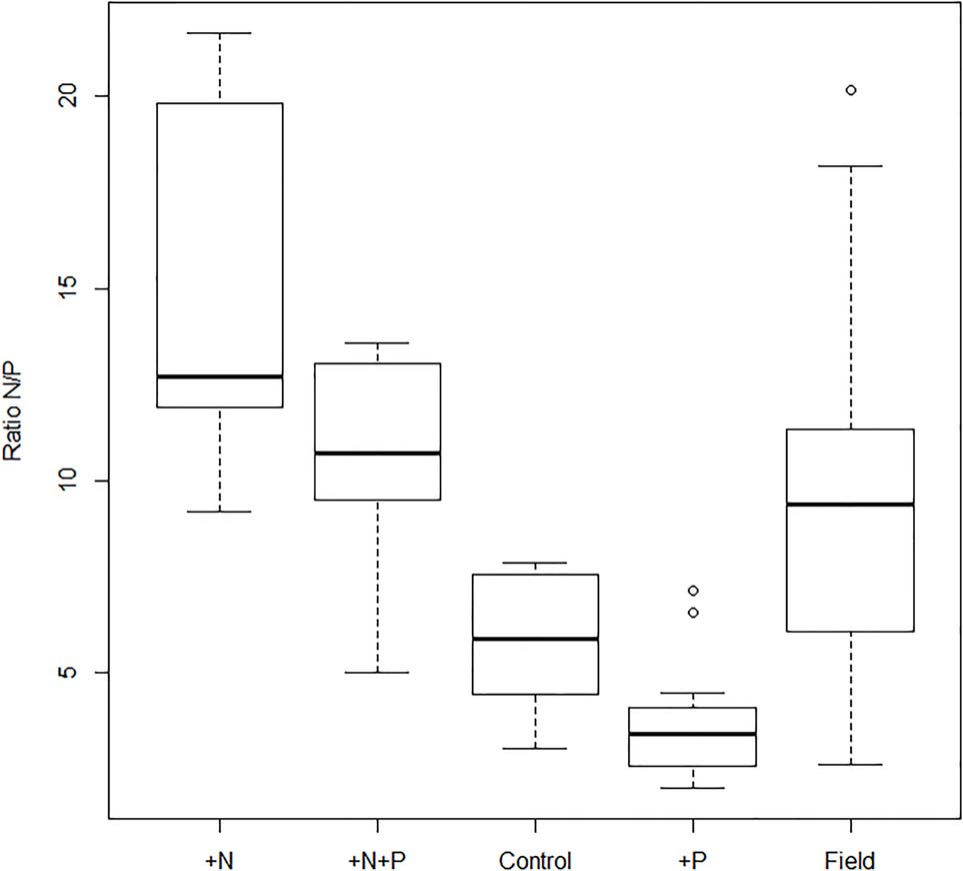
Leaf N:P ratios of plants in the different fertilization treatments and plants sampled in nature. Sample sizes were as follows: plants in the control (*N* = 9), +P (*N* = 11), +N (*N* = 11), +N+P (*N* = 11), and plants sampled in nature (*N* = 36). For the analysis of leaf N:P ratio according to plant fertilization treatment, all comparisons were significant (Tukey’s tests; *p* < 0.01) except plants in the control and +P treatments that showed similar N:P ratios (Tukey’s test; *p* = 0.47). The results from the Wilcoxon pairwise comparisons showed that the N:P ratio of plants sampled in nature were significantly different from plants in the control, +P, and +N treatments (*p* < 0.05) and overlapped with the N:P ratio of plants in the +N+P treatment (*p* = 0.17). The horizontal line corresponds to the median of leaf N:P ratios. The bottom and top of the box correspond to the 25th and 75th percentiles, respectively.

Plant fertilization treatments induced the expected variation in leaf stoichiometry; nettles varied in leaf N:P ratio among the four fertilization treatments (F_3,38_ = 28.92, p < 0.001, Fig 1), and the leaf N:P ratio in each fertilization treatment was constant through time. Leaf N:P ratio was highest for plants in the +N treatment, followed by plants in the +N+P treatment and comparably lower in the control and +P treatments (Fig 1). Plants in the field had similar N:P ratio to plants in the +N+P treatment (Fig 1). This result shows that leaf N:P ratios of fertilized plants were within the range of variation of plants found in the field. This was also true for the absolute values of nitrogen and phosphorus, but they were in the upper range of the natural variation.

Looking at plant absolute nutrient content between fertilization treatments, we found that plants in +N and +N+P treatments contained the same amount of nitrogen and significantly more than plants in the control and +P treatments (S1 Appendix). Similarly, plants fertilized with only phosphorus contained a significantly higher amount of phosphorus in their leaves than the other plants (S1 Appendix). On the other hand, the relationship between the absolute amount of phosphorus in the fertilizer and leaf phosphorus content was more complex. Plants in the +N+P treatment contained a lower amount of phosphorus in their leaves than plants in the +P treatment even though the absolute amount of phosphorus received was the same (S1 Appendix). We think that this discrepancy is likely to be explained by the fact that nitrogen stimulated plant growth (personal observation) and resulted in a higher dilution of the phosphorus for those plants. Nevertheless, no quantification of plant biomass was done to corroborate this hypothesis.

### Insect stoichiometry

**Fig 2:**
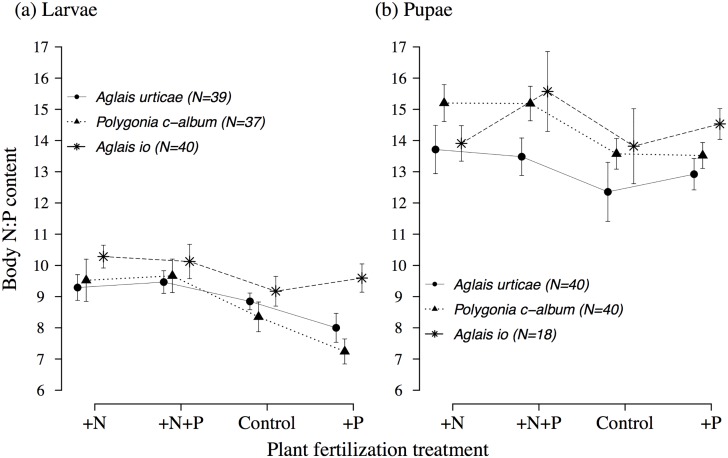
Body N:P ratios in fifth-instar larvae (a) and pupae (b) for *Algais urticae*, *Polygonia c-album*, and *Aglais io* (mean±se) according to plant fertilization treatment.

At the fifth-instar larval stage, both plant fertilization treatment and species explained variation in body N:P ratio (Table 1, Fig 2a). Overall, larvae in the +N and +N+P treatments showed significantly higher body N:P ratio than larvae feeding on the +P treatment (Tukey’s tests; *p* < 0.01). Moreover, *A. io* larvae had significantly higher body N:P ratio than *P. c-album* and *A. urticae* (Tukey’s test; *p* < 0.01). At the pupal stage, plant fertilization treatment, species, and start date explained variation in body N:P ratio (Table 1). Body N:P ratio slightly increased through time (estimate = +0.15±0.07). *P.c.album* showed a significantly higher body N:P ratio than *A.urticae* pupae (Tukey’s test; *p* < 0.01, Fig 2b), however there were no significant differences observed between plant fertilization treatments from the post-hoc tests.

**Table 1:** Analysis of deviance table (type II) showing the effect of fertilizer, species, and start date on body N:P ratios in fifth-instar larvae and pupae.

Body N:P ratio	*Fifth-instar larvae*				*Pupae*			
	*df*	Sum Sq	F	*p*	*df*	Sum Sq	F	*p*
Fertilizer	3	48.1	7.6	**< 0.001**	3	39.5	3.4	**0.02**
Species	2	27.1	6.4	**< 0.01**	2	47.1	6.2	**< 0.01**
Start date	1	-	-		1	19.2	5.0	**0.02**
Residuals	110	231.6	-	-	91	348.1	-	-

The analyses done on absolute amount of nitrogen showed that for both specialist species, *A. urticae* and *A. io*, body content of nitrogen in fifth-instar larvae varied with plant fertilization treatment (S2 Appendix, S1a Fig). Body content of nitrogen was highest for larvae of the two specialist species feeding on the +N and +N+P treatments, which also corresponded to larvae fed with plants with the highest absolute amount of nitrogen (S1 Appendix, S2 Appendix). We did not find any significant differences between plant fertilization treatments for *P. c-album* larvae (S2 Appendix). The higher variance in body content of nitrogen among fifth-instar larvae in *P. c-album* may explain why we did not find a significant effect of plant fertilization treatment in this species (S1a Fig). At the pupal stage, it seems that the specialist species compensated for the discrepancy in body content of nitrogen between plant fertilization treatments that were observed in fifth-instar larvae (S3 Appendix, S1b Fig). In *A. urticae* and *A. io*, pupal body content of nitrogen did not vary significantly between plant fertilization treatments. We still found an interaction between fertilizer and species (S3 Appendix), which is explained by the response of the generalist species. In *P. c-album*, we found a lower pupal body content of nitrogen in the +P treatment compared to pupae of the other species on the same treatment (S1b Fig).

Body content of phosphorus varied between developmental stages and species but was not sensitive to plant fertilization treatment (S2 Text, S3 Appendix, S1c and S1d Fig). Fifth-instar larvae contained significantly more phosphorus than pupae. Moreover, we observed a species-specific response in pupae. *A. urticae* retained significantly higher amounts of phosphorus than *A. io* and *P. c-album* pupae (S1d Fig).

### Larval performance

Survival rate did not vary according to plant fertilization treatment but varied between species (*χ* ^2^ = 123.18, *df* = 2, *p* < 0.001). Survival was highest for *P. c-album* larvae, followed by *A. urticae*, and last *A. io* (survival rates were 78.2±6.5, 34.3±3.6, 15.5±7.4, mean % ±se, respectively).

**Fig 3:**
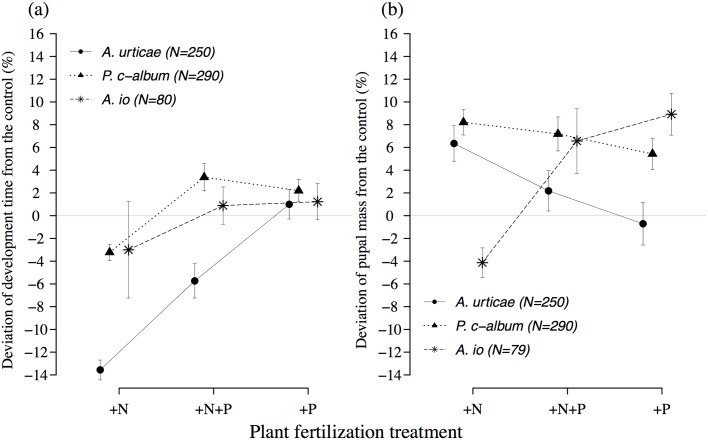
Deviation of development time from the control (% ± se) (a) and deviation of pupal mass from the control (% ± se) (b), for *Aglais urticae*, *Polygonia c-album*, and *Aglais io* larvae according to plant fertilization treatment.

In *A. urticae*, larvae feeding on nitrogen-fertilized plants had their development shortened by 1.5 to 3.6 days, which corresponds to a decrease of 5.7 to 13.6% compared to larvae feeding in the control treatment (Table 2, Fig 3a). Plant fertilization treatment also induced variation in pupal mass (Table 2). Larvae in the +N treatment became significantly heavier than larvae feeding on the other treatments (Fig 3b). Note that these positive responses in terms of larval performance to the +N treatment (and to a lesser extent the +N+P treatment) compared to the control and +P treatments corresponded to larvae feeding on plants with the highest N:P ratio (Figs 1 and 3). This also corresponded to plants with the highest absolute amount of nitrogen (S1 Appendix).

**Table 2:** Analysis of deviance table (type II Wald Chi-square test and type III Wald Chi-square test when a model with an interaction term has been selected). Values represent the effect of fertilizer, sex, their interaction, and start date on the deviation of development time to pupation (DT) and pupal mass from the control treatment for *Aglais urticae*, *Polygonia c-album*, and *Aglais io*.

		*A. urticae*				*P. c-album*				*A. io*			
		DT		pupal mass		DT		pupal mass		DT		pupal mass	
	*df*	Chisq	*p*	Chisq	*p*	Chisq	*p*	Chisq	*p*	Chisq	*p*	Chisq	*p*
Fertilizer	3	119.3	**< 0.001**	17.3	**< 0.001**	37.4	**< 0.001**	20.8	**< 0.001**	-	-	28.4	**< 0.001**
Sex	1	-	-	-	-	4.8	**0.03**	2.7	0.10	15.5	**< 0.001**	48.3	**< 0.001**
Start date	1	3.7	0.05	-	-	-	-	10.4	**0.001**	-	-	-	-
Fertilizer:Sex	3	-	-	-	-	-	-	7.8	0.05	-	-	-	-

*Note*: Fertilizer, sex, and start date were considered as fixed effects of the models. Family or clutch were considered as a random effect of the mixed models (see text for more details). For size models, Akaike Information Criterion (AIC) values were calculated using maximum likelihood (ML) but other parameters were from REML estimation.

In contrast, *P. c-album* and *A. io* showed a weaker response to plant fertilization treatment. *P. c-album* larvae did show a faster development (+3.2%) when feeding on +N treatment compared to larvae feeding on the other treatments (Table 2, Fig 3a), but to a lower extent. In *P. c-album*, there was also a significant interaction between plant fertilization treatment and sex for pupal mass (Table 2). In *A. io*, development time did not vary according to plant fertilization treatment but did vary between sexes (Table 2). Interestingly, for this species larvae reached a higher pupal mass on the +P treatment, which corresponded to plants with the lowest N:P ratio (Figs 1 and 3b). This also corresponded to plants with the highest absolute amount of phosphorus (S1 Appendix). The significant effect of sex in the models of performance selected for both *P. c-album* and *A.io* is likely to be caused by the sex dimorphism in these species. As a result of their smaller size, males had a shorter time to pupation.

## Discussion

We found that the fertilization treatments induced the expected differences in plant stoichiometry. Both types of fertilization treatment (nitrogen and phosphorous) influenced plant N:P ratio. Plants fertilized with nitrogen had a higher N:P ratio than plants not fertilized with nitrogen. Conversely, phosphorus fertilization induced a decrease in N:P ratio. *U. dioica* is known to thrive in high-nutrient conditions, and the leaf content analyses showed that plant phenotype indeed responded to nutrient input. Moreover, N:P ratio of leaves in the fertilization treatments completely overlapped with the natural range found in plants collected in the field, but were in the upper range of the natural variation in terms of absolute amount in nitrogen and phosphorus. Thus, the larval rearing experiment can be seen as a test of larval ability to respond to the natural variation in their food stoichiometry, but to some extent also of their ability to take advantage of extra nutrients.

Larval and pupal stoichiometry largely matched that of their food, indicating that there was no obvious nutrient limitation that lead to nutrient imbalance in any of the treatments (Fig 2, Table 2). However, it is interesting to note that the response to plant fertilization treatment in terms of body content of nitrogen in fifth-instar larvae was weaker for the generalist *P. c-album* than in the two specialists (largely caused by a much higher variance). This could indicate a higher level of adaptation of the specialists to their single host plant, which would be in line with our main hypothesis.

Larval survival rate was not affected by plant fertilization treatment but varied considerably between species. However, these species-specific responses should be interpreted with caution, since they are likely to be highly affected by the rearing method. In order to maintain individual control, larvae had to be reared singly. It is known to adversely affect survival of *A. urticae* and *A. io*, which normally feed gregariously. Moreover, neonate larvae of *A. urticae* and *A. io* are very small compared to *P. c-album*, which make them more sensitive to initial feeding position (and individual leaf quality). In nature, *A. urticae* and *A. io* females are much more discriminating in their selection of oviposition site, and preferentially lay their eggs on the apical leaves of the plant [43]. As a consequence, the high overall mortality of the two clutch layers are likely to be caused by our inability to provide the larvae with optimal conditions for larval establishment and feeding, but we do not see any reason why this high mortality should be linked to the differences in larval performance observed between fertilization treatments (as mortality did not vary according to plant fertilization treatment).

The plant fertilization treatment affected larval development and pupal mass of all three butterfly species, although in somewhat different ways. Both *P. c-album* and *A. urticae* developed faster on plants in the +N treatment, which also corresponded to plants with the highest N:P ratio. Interestingly, *A. urticae* also reached a larger size on this treatment, in spite of a shorter development time. The response of *P. c-album* was of a smaller magnitude, and development time was only affected at the highest N:P ratio (Fig 3a). The response of *A. urticae* seemed more directly tuned to the N:P ratio of their food, as pupal mass increased and development time decreased proportionally to plant N:P ratio (Figs 1 and 3). This difference in sensitivity to nitrogen also appears to support the hypothesis that nettle-specialists should be better adapted to make use of the extra nutrient input provided by nettles growing under unusually nitrogen-rich conditions.

On the other hand, the other specialist in the study, *A. io*, did not respond to plant fertilization treatment in terms of development time, but did so for pupal mass. Moreover, larvae reached the largest size on the +P treatment, which also corresponded to plants with the lowest N:P ratio. Thus, while pupal mass in *A. urticae* (and to a lesser extent *P. c-album*) was positively affected by nitrogen, pupal mass of *A. io* were positively affected by phosphorus. If the differences between *A. urticae* and *P. c-album* are to be attributed to their differences in host plant specialization, then this is contradicted by the divergent response of *A. io*. The sample size for *A. io* was substantially smaller than of the other two species, but this can hardly be the sole explanation for this deviation.

A possible alternative explanation comes from the observation that *A. urticae* showed a clearer positive response to food stoichiometry than the other two species, and that this was more pronounced for development time than it was for pupal mass. *A. urticae* is the only species in our study that is bivoltine in southern Sweden, and thus is under time pressure to be able to complete two full generations during the summer. It has been shown previously that breeding success in *A. urticae* is sensitive to changes in leaf quality (water content and nitrogen levels), especially for the second summer generation [44, 45]. In comparison, the two univoltine species are unable to fit a second generation into the favorable season at these latitudes, but they have ample time for one generation [37, 46]. As a consequence, *A. urticae* should be more dependent on nutrient-rich food, and should also be expected to favor shorter development times over large adult size, compared to the two univoltine species. In other words, the differences between the species may be better explained by life cycle differences (voltinism) than degree of specialization.

Surprisingly, we did not observe a consistent effect of phosphorus on the larvae. Following the Growth Rate Hypothesis and other studies done on insects [2, 13], the bivoltine *A. urticae* should be expected to respond to increased phosphorus levels by speeding up development. Instead we found that larvae of the univoltine *A. io* were the only species to respond positively to phosphorus, even though larvae are not under strong time pressure to develop. Our results, at the scale of the individual, also contrast with population-level studies, which found that phosphorus can be a limiting resource with a potential to impair population growth [4, 17]. Our results seem to indicate that, for our study species, phosphorus was not limiting.

Still, the reason for this lack of a phenotypic response to phosphorous in our study species is somewhat puzzling. Frost and Elser [47] have also found that phosphorus affected larval feeding rates in mayfly nymphs negatively. They suggested a regulatory feeding mechanism to explain lower growth in nymphs feeding on food with lower C:P ratio. Larvae fed with unusually P-rich food might reach their phosphorus requirements faster and the need to eliminate excess phosphorous may reduce larval feeding rates. In our study, larvae might have had to deal with phosphorus excess. Even if N:P ratios in our study were overlapping with ratios found in the field, the absolute amounts of phosphorus were in the upper range. This might, in turn, suggest that both stoichiometry and the absolute amount of nutrients are relevant for understanding our results. Further studies on adult egg-laying preference on nitrogen—and/or phosphorus—rich plants would be useful in order to further investigate the consequences of food stoichiometry and to understand the factors determining host plant quality for insects. This may in turn explain their pattern of distribution, especially for those species that show substantial niche overlap.

In summary, our study highlights that species with different life histories may respond differently to changes in food stoichiometry. This study is unusual in that we were able to investigate several species feeding on the same plants while differing in life history. Even so, it is hard to draw any strong general conclusions based on a three-species comparison only. Still, we find it likely that some of the species-specific responses to variation in food stoichiometry are linked to differences in life history. Our hypothesis that specialists should be more sensitive than generalists to intraspecific variation in nutrient content was supported by a difference in performance between *A. urticae* and *P. c-album*. However, the deviating performance of the specialist *A. io* weakens the argument for a specialist adaptation to plant stoichiometry. In *A. io*, pupal mass showed an opposite pattern in its sensitivity to N:P ratio and we did not observe differences in development time between food treatments. An alternative explanation for the observed differences may instead be attributed to their differences in life cycle. The time pressure to accomplish two reproductive cycles in a relatively short period of time (the Swedish summer) for *A. urticae* may participate to explain its larger sensitivity to high N:P ratios and the apparent inconsistency between the two specialist species. The need for a shorter development time may make the adults more prone to seek out and lay their eggs on nettles with higher nitrogen content. Moreover, as a consequence of the bivoltine life cycle of *A. urticae*, individuals need to invest more into reproductive tissues which has a cost in terms of nitrogen [48, 49].

Although stoichiometry can explain variation in life-history traits [7], our study suggests that variation in life history can also influence how species respond to stoichiometric differences [50]. With increased levels of nutrient leakage from human activities, it is important to understand the responses of plants and the animals that feed on them. A negative correlation between the level of eutrophication and butterfly diversity has already been reported [51]. In addition, although the geographic range expansion following global warming is typically more pronounced in generalists than in specialists, the ability to take advantage of a high-nutrient resource such as nettles may have facilitated geographic range expansion of nettle-specialists [34]. Importantly, the responses to stoichiometry may interact with effects of climate warming to alter patterns of growth and development. For example, such an interaction may further increase the incidence of bivoltinism in temperate insects [52, 53], which may have significant consequences for both natural and agricultural ecosystems.

## Supporting Information

S1 Text Statistical Analyses. (PDF)

S2 Text Effect of plant fertilization treatment on body content of phosphorus in fifth-instar larvae. (PDF)

S1 Appendix Effect of plant fertilization treatment on leaf absolute nitrogen and phosphorus content. (PDF)

S2 Appendix Effect of plant fertilization treatment on body content of nitrogen in fifth-instar larvae. (PDF)

S3 Appendix Effect of plant fertilization treatment on body content of nitrogen in pupae. (PDF)

S4 Appendix Effect of plant fertilization treatment on body content of phosphorus in pupae. (PDF)

S1 Fig Effect of plant fertilization treatment on body content of nitrogen and phosphorus in fifth-instar larvae (respectively (a) and (c)) and pupae ((b) and (d)) for *Algais urticae*, *Polygonia c-album*, and *Aglais io* (mean±se). (PDF)
