# Supplementary material for: Plant Fertilization Interacts with Life History: Variation in Stoichiometry and Performance in Nettle-Feeding Butterflies
Source: PLoS One. 2015 May 1;10(5):e0124616. doi: 10.1371/journal.pone.0124616 (PMC4416804; doi:10.1371/journal.pone.0124616)
Supplement: S1 Text — (PDF) [file pone.0124616.s001.pdf]

S1 Text. Statistical Analyses.

In consistency with the manuscript, all statistical analyses presented in the Supporting Information were performed in the statistical program R, version 3.0.2 [1]. Model fitting selected for the fixed factors following a backward elimination procedure. Models comparison was performed using the anova function and the Akaike Information Criterion (AIC) as a guideline. The fit of the models retained was estimated according to the distribution of the residuals.

References

[1] R Development Core Team (2013) *R: A Language and Environment for Statistical Computing. Version 3.0.2*. The R Foundation for Statistical Computing, Vienna, Austria.
