# Supplementary material for: Plant Fertilization Interacts with Life History: Variation in Stoichiometry and Performance in Nettle-Feeding Butterflies
Source: PLoS One. 2015 May 1;10(5):e0124616. doi: 10.1371/journal.pone.0124616 (PMC4416804; doi:10.1371/journal.pone.0124616)
Supplement: S2 Text — (PDF) [file pone.0124616.s002.pdf]

S1 Appendix. Effect of plant fertilization treatment on leaf absolute nitrogen and phosphorus content.

We analyzed the effect of fertilization treatment (Control, +P, +N, +N+P) on leaf absolute nitrogen and phosphorus content independently. Differences in plant %N and %P were tested using ANOVAs followed by pairwise t-tests for paired comparisons with Holm-Bonferroni correction. We tested for the effect of plant fertilization treatment, time, and the interaction between these two variables. Time corresponds to the plant sampling date. We included time as a covariate to investigate if plant nutrient content varied with time.

The model selected for leaf absolute nitrogen content only retained the effect of plant fertilization treatment ($F_{3,38}$=20.3, $p< 0.001$, $R^2{}_{adjusted}$=58.5). Almost all comparisons were significant except plants in the control and +P treatments, and plants in the +N and +N+P treatments (Table A, Fig. A, left).

The model selected for leaf absolute phosphorus content retained the interaction between plant fertilization treatment and time. A follow-up analysis on the effect of time on each of the plant fertilization treatment showed that only plants in the +N treatment significantly varied in phosphorus content through time and this correlation was negative (estimate= -0.01, t=-2.7, p=0.02). Plants in the +N treatment corresponded to plants with the lowest amount of phosphorus and this amount decreased through time. Therefore the relative position of the four fertilization treatments in terms of leaf absolute phosphorus content was kept through time. For this reason, we chose not to consider this interaction as it will not affect the between-groups comparisons (of main interest here). Thus, the

final model only retained the effect of plant fertilization treatment ($F_{3,38}$=12.8, $p < 0.001$, $R^2_{adjusted}$=46.3). Almost all comparisons were significant except plant in the control and +N+P treatments, and the difference between plants in the +N and +N+P treatments was close to significance (Table A, Fig. A, right).

Table A: Elemental content (% nitrogen, and % phosphorus) of plants in each fertilization treatment and plants from the field (mean ± sd). N corresponds to the number of plants used for each analysis.

Nutrient content /treatment	Control (N=9)	+P (N=11)	+N (N=11)	+N+P (N=11)	Field (N=36)
%nitrogen	4.1±1.0	3.5±1.3	7.3±1.6	7.1±1.6	3.8±1.0
%phosphorus	0.75±0.23	0.96±0.19	0.51±0.12	0.70±0.16	0.53±0.33

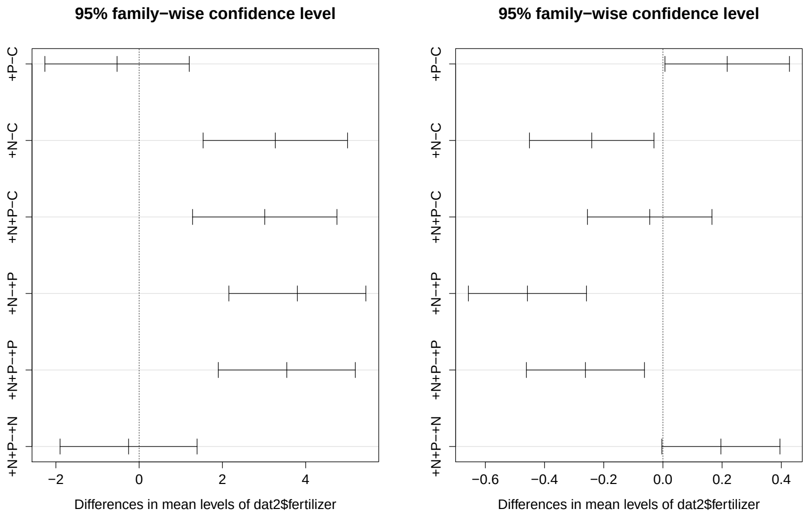

Figure A: Visual representation of the results of the Tukey's tests comparing the absolute nitrogen (left) and phosphorus (right) content between plant fertilization treatments (C-control, +P,+N+P,+N).
