# Supplementary material for: Plant Fertilization Interacts with Life History: Variation in Stoichiometry and Performance in Nettle-Feeding Butterflies
Source: PLoS One. 2015 May 1;10(5):e0124616. doi: 10.1371/journal.pone.0124616 (PMC4416804; doi:10.1371/journal.pone.0124616)
Supplement: S1 Appendix — (PDF) [file pone.0124616.s003.pdf]

S2 Appendix. Effect of plant fertilization treatment on body content of nitrogen in fifth-instar larvae.

We investigated the effect of plant fertilization treatment on body content of nitrogen in fifth-instar larvae using ANOVAs followed by Tukey's tests. The effect of plant the fertilization treatment on body content of nitrogen in *P. c-album* fifth-instar larvae was analyzed separately from the other two species because of the much higher variance in the response for this species (no transformation was possible as an alternative). Thus, we built two separate models; one for the two specialist species (*A. urticae* and *A. io*) and one for *P. c-album.* In the first model, we tested for the effect of species, plant fertilization treatment, their interaction, and we included start date as a covariate. In the second model we tested for the effect of plant fertilization treatment and start date.

The model selected for body content of nitrogen in fifth-instar larvae for the two specialist species (*A. urticae* and *A. io*) retained the effect of species and plant fertilization treatment. All mutliple comparisons from the Tukey's tests were significant except between plants in the +N and +N+P treatments. The final model is presented in Table A and results are shown in Fig. S1a.

We did not find a significant effect of plant fertilization treatment on fifth-instar larvae body content of nitrogen in *P. c-album.* The final model selected was the null model. Fig. S1a illustrates the results.

Table A: Type II ANOVA table showing the effect of species and plant fertilization treatment on body content of nitrogen in fifth-instar larvae of the two specialist species (*A. urticae* and *A.io*).

Fifth-instar larvae body content of nitrogen	Sum Sq	df	F	*P*
Species	9.30	1	15.5	<0.001
Fertlizer	39.68	3	22.1	<0.001
Residuals	41.88	70		
