# Supplementary material for: Plant Fertilization Interacts with Life History: Variation in Stoichiometry and Performance in Nettle-Feeding Butterflies
Source: PLoS One. 2015 May 1;10(5):e0124616. doi: 10.1371/journal.pone.0124616 (PMC4416804; doi:10.1371/journal.pone.0124616)
Supplement: S2 Appendix — (PDF) [file pone.0124616.s004.pdf]

S3 Appendix. Effect of plant fertilization treatment on body content of nitrogen in pupae.

We investigated the effect of plant fertilization treatment on body content of nitrogen in pupae using an ANOVA. We tested for the effect of species, plant fertilization treatment, their interaction, and we included start date as a covariate. The final model selected is presented in Table A and results are shown in Fig. S1b.

Table A: Type III ANOVA table showing the effect of species, plant fertilization treatment, their interaction, and start date, on body content of nitrogen in pupae for the three species (*A. urticae*, *A.io* and *P. c-album*).

Pupae body content of nitrogen	Sum Sq	df	F	*P*
Intercept	43.96	1	191.5	<0.001
Species	4.62	2	10.1	<0.001
Fertilizer	1.82	3	5.0	0.055
Start date	6.40	1	27.9	<0.001
Species:Fertilizer	3.07	6	2.2	0.048
Residuals	19.52	85		
