# Supplementary material for: Plant Fertilization Interacts with Life History: Variation in Stoichiometry and Performance in Nettle-Feeding Butterflies
Source: PLoS One. 2015 May 1;10(5):e0124616. doi: 10.1371/journal.pone.0124616 (PMC4416804; doi:10.1371/journal.pone.0124616)
Supplement: S3 Appendix — (PDF) [file pone.0124616.s005.pdf]

S2 Text. Effect of plant fertilization treatment on body content of phosphorus in fifth-instar larvae.

We investigated the effect of plant fertilization treatment on body content of phophorus in fifth-instar larvae using an ANOVA. The response variable was log-transformed to meet the assumptions of the ANOVA. We tested for the effect of species, plant fertilization treatment, their interaction, and we included start date as a covariate. The final model selected was the null model. Fig. S1c illustrates the results.
