# Supplementary material for: Plant Fertilization Interacts with Life History: Variation in Stoichiometry and Performance in Nettle-Feeding Butterflies
Source: PLoS One. 2015 May 1;10(5):e0124616. doi: 10.1371/journal.pone.0124616 (PMC4416804; doi:10.1371/journal.pone.0124616)
Supplement: S4 Appendix — (PDF) [file pone.0124616.s006.pdf]

S4 Appendix. Effect of plant fertilization treatment on body content of phosphorus in pupae.

We investigated the effect of plant fertilization treatment on body content of phophorus in pupae using an ANOVA. The response variable was log-transformed to meet the assumptions of the ANOVA. We tested for the effect of species, plant fertilization treatment, their interaction, and we included start date as a covariate. The final model selected is presented in Table A and the results are shown in Fig. S1d.

Table A: Type II ANOVA table showing the effect of species on body content of phosphorus in pupae for the three species (*A. urticae*, *A.io* and *P. c-album*).

Logarithm of pupae body content of phosphorus	Sum Sq	df	F	*P*
Species	0.32	2	11.21	<0.001
Residuals	1.37	96		
