# Supplementary material for: Plant Fertilization Interacts with Life History: Variation in Stoichiometry and Performance in Nettle-Feeding Butterflies
Source: PLoS One. 2015 May 1;10(5):e0124616. doi: 10.1371/journal.pone.0124616 (PMC4416804; doi:10.1371/journal.pone.0124616)

S1 Figure. Effect of plant fertilization treatment on body content of nitrogen and phosphorus in fifth-instar larvae (respectively (a) and (c)) and pupae (respectively (b) and (d)) for *Algais urticae*, *Polygonia c-album*, and *Aglais io* (mean±se).

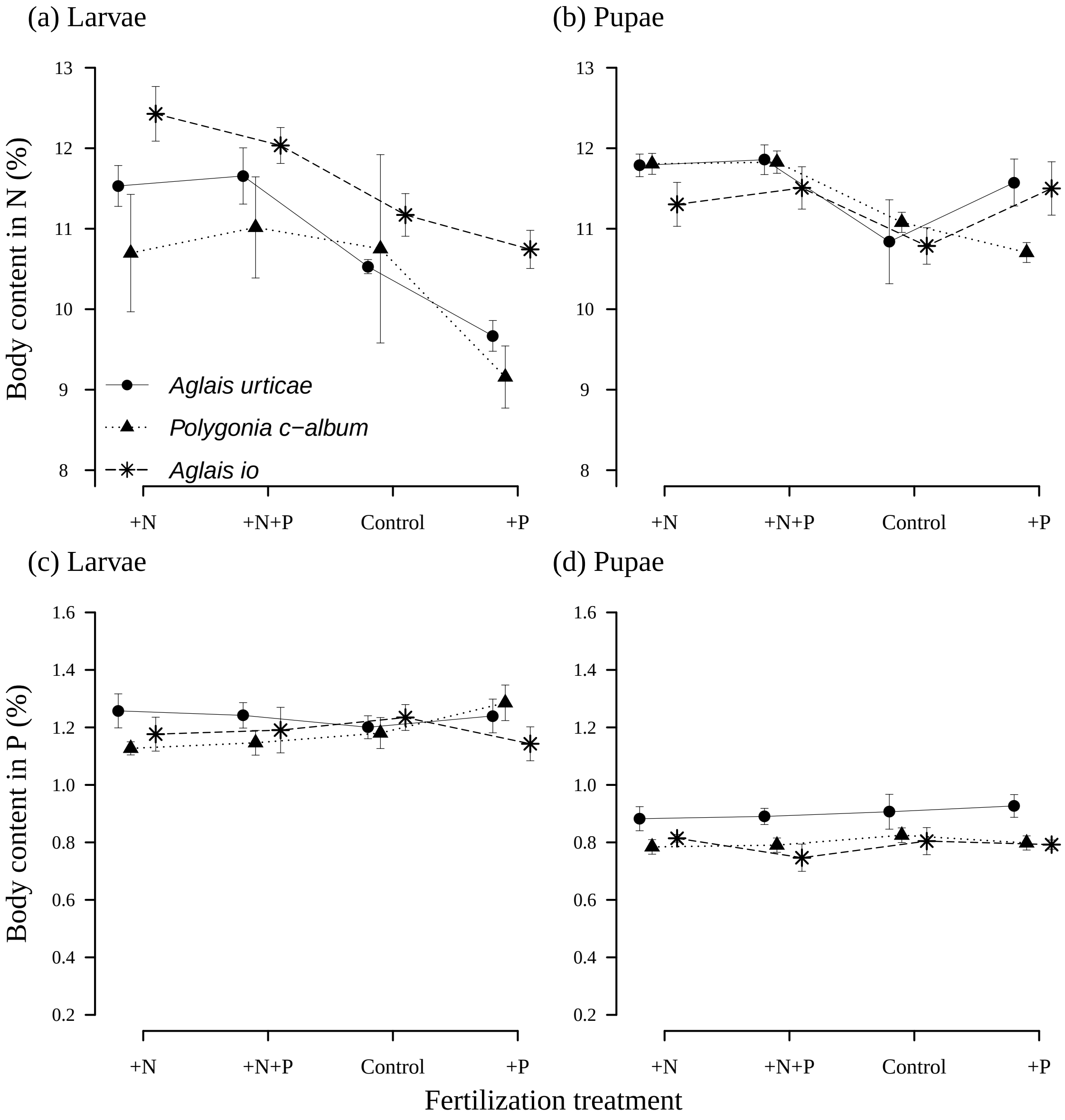

Supplement: S1 Fig — (PDF) [file pone.0124616.s007.pdf]
